# Peer Review of “COVID-19 Return to Sport: NFL Injury Prevalence Analysis”

**DOI:** 10.2196/38730

**Published:** 2022-04-22

**Keywords:** COVID-19, sport, injury, prevalence, cause, data, statistics, pain, training, practice, physiology, adaptation

*This is a peer-review report submitted for the paper “COVID-19 Return to Sport: NFL Injury Prevalence Analysis”*

## Round 1

### General Comments

This paper [1] compares the number of injuries in the National Football League between 3 consecutive years to analyze the impact of the COVID-19 pandemic. Although the paper is investigating an interesting subject, I believe it lacks enough analysis and final conclusion. One important addition to this paper could be an analysis of the differences between the hours of exercise in those 3 years. With the current paper, we do not know if there was actually a decrease in the number of hours each player exercised. This extra analysis will help to understand if the increase in injuries were really resulted from less exercise or if it was because of health issues such as mental and physical problems caused by the pandemic.

### Specific Comments

1. Please define resistance exercise and postresistance exercise.
2. Please refrain from citing a figure in the abstract.
3. Why didn't you include sick days in your analysis?
